# A Comprehensive Review of the Harmful Compounds in Electronic Cigarettes

**DOI:** 10.3390/toxics13040268

**Published:** 2025-03-31

**Authors:** Eduard Ferney Valenzuela Toledo, Ivana Ferreira Simões, Marcel Tavares de Farias, Lucas Almir Cavalcante Minho, Jaquelide de Lima Conceição, Walter Nei Lopes dos Santos, Paulo Roberto Ribeiro de Mesquita, Aníbal de Freitas Santos Júnior

**Affiliations:** 1 Department of Life Sciences, State University of Bahia (UNEB), Salvador 41195-001, Bahia, Brazil; efvalto@gmail.com (E.F.V.T.); ivanafs99@gmail.com (I.F.S.); farias.mt@gmail.com (M.T.d.F.); jaquelidelima@gmail.com (J.d.L.C.); waltrs8@gmail.com (W.N.L.d.S.); 2 Institute of Chemistry, Federal University of Bahia (UFBA), Salvador 40170-115, Bahia, Brazil; cavalcante.lpi@gmail.com; 3 Secretariat of Agriculture, Livestock, Irrigation, Fisheries and Aquaculture of the Government of the State of Bahia (SEAGRI), Agricultural Technology Center of the State of Bahia, Salvador 40170-110, Bahia, Brazil; prrmesquita@gmail.com

**Keywords:** e-cigarette, chemical analysis, harmful compounds, toxicological impact

## Abstract

Electronic cigarettes (e-cigarettes) are devices designed to vaporize a liquid solution, offering an alternative to traditional tobacco consumption. The identification, detection, and analysis of the compounds present in these devices are crucial for understanding their impacts on health and the environment. Numerous studies have identified a diverse range of compounds emitted by e-cigarettes, including well-known substances such as nicotine, thermal degradation products, and other toxicants that may be harmful or carcinogenic. Although e-cigarettes are often considered an alternative to conventional smoking, they are not without risks. Recent research has increasingly focused on assessing the health impacts of e-cigarettes, integrating findings from various scientific disciplines. Two primary analytical approaches are used for the sample preparation, identification, and quantification of these compounds. The first approach focuses on aerosol analysis, utilizing techniques such as headspace static extraction and gas chromatography coupled with mass spectrometry (GC-MS). The second approach is directed towards liquid analysis, employing liquid–liquid extraction techniques and liquid chromatography (LC) systems. Given the constant publication of new research in this area, a comprehensive review that consolidates information on identified compounds, sample preparation methods, and extraction and analysis techniques is necessary to integrate current knowledge and address emerging findings.

## 1. Introduction

Tobacco, native to the Americas, has been cultivated and utilized by various indigenous cultures for centuries. Tobacco leaves contain various chemical compounds that, when burned, release nicotine, an addictive alkaloid, along with other thermal degradation products [1]. Tobacco contains numerous chemical compounds, including nicotine, minor alkaloids, sugars, proteins, and several additives. The combustion of tobacco generates thousands of degradation products, many of which are toxic and carcinogenic [2]. These products include carbonyl compounds, mono and polycyclic aromatic hydrocarbons (PAHs), tobacco-specific nitrosamines (TSNAs), and potentially toxic metals (PTMs) [3,4,5]. The specific composition of tobacco and tobacco products varies depending on the type of product and the manufacturing processes used.

Tobacco is consumed primarily in two forms: smoked (cigarettes, cigars, pipes) and smokeless (snuff, chewing tobacco). Smoking tobacco is the most common form and is associated with the inhalation of a complex mixture of toxic chemicals. Tobacco use is strongly associated with a variety of health risks, including cardiovascular diseases, respiratory diseases, and various types of cancer. Both smoked and smokeless tobacco products release toxic and carcinogenic compounds that can cause significant damage to the organs and systems of the human body. The risks include congenital defects in the offspring of smoking mothers, as well as increased mortality from cardiovascular diseases and cancer [6,7,8].

In response to the idea of reducing the negative impacts of tobacco, the industry diversified its products to include the now-famous electronic cigarettes [9]. Electronic cigarettes, also known as vaping devices, e-cigs, e-hookahs, mods, vape pens, vaporizers, tank systems, or nicotine delivery systems, are portable, battery-powered vaporizing devices that simulate smoking by heating a liquid to produce an inhalable aerosol (vapor), known as e-liquid. The liquid, also known as ’e-liquid/e-juice’, typically contains a mixture of propylene glycol (PG), glycerin (GLY), flavorings, nicotine in varying concentrations, and unusual additives such as tetrahydrocannabinol (THC) or cannabinoid oils (CBD) [10,11].

The debate over the effectiveness of vape devices, or e-cigarettes, as a tool for smoking cessation has been intense and complex. While some studies suggest they may be beneficial for certain individuals in their efforts to quit smoking [12], other evidence indicates that vape devices can have counterproductive effects and, in some cases, may induce nicotine addiction rather than eliminate it [13]. Presently, there is no electronic device that has been endorsed by a health institution or proven to have therapeutic benefits for counteracting addiction or other health issues [14]. In this sense, many young people mistakenly believe that e-cigarettes can help them overcome other addictions and that they are less risky [15,16]. This misconception highlights the need for greater awareness and education about the effects of vaping. It is important to spread accurate information to prevent the spread of these harmful myths.

Like traditional cigarettes, vape devices contain nicotine, a highly addictive substance. The nicotine concentration in vape liquids can vary, meaning users may be exposed to significant levels of this substance, potentially contributing to addiction. According to a study conducted in the United States, the average nicotine concentration in e-cigarettes sold has increased across all flavor categories and for rechargeable e-cigarettes [17]. The proportion of total dollar sales for higher-nicotine-concentration e-cigarettes (>4% mg/mL) rose from 12.3% to 74.7%, with a similar rise in unit share. Products with zero nicotine accounted for less than 1% of the dollar market share throughout the period analyzed. Another study found that fruity, menthol, and mint concept flavor e-cigarettes experienced the largest increase, while tobacco concept flavors and pod-based concept flavor e-cigarettes dominated sales [18]. Additionally, menthol-flavored e-cigarettes have recently been approved by the FDA (Food and Drug Administration) [19]. This variety of devices has increased sales drastically. According to the latest research study, the global e-cigarette market is projected to grow from USD 22.5 billion in 2022 to USD 47.5 billion in 2028 at a compound annual growth rate (CAGR) of 13.5% during the forecast period 2023 to 2028 [20].

The availability of appealing flavors in vape liquids, combined with targeted marketing strategies, makes these devices particularly enticing to young people, facilitating the initiation of nicotine use [21]. From an environmental perspective, disposable devices and e-liquids contribute to electronic and chemical waste pollution, as batteries and components do not degrade easily, potentially impacting environmental components such as water, air, and soil [22,23]. Regarding health, vape liquids contain potentially toxic compounds that may damage the lungs and other organs. Furthermore, the long-term effects of vapor exposure remain not fully understood, particularly in adolescents whose bodies are still developing.

Instrumental techniques are crucial for detecting and quantifying substances present in e-cigarettes, as they provide precise and reliable data on the chemical composition of vape liquids. Separation techniques such as gas chromatography–mass spectrometry (GC-MS) and high-performance liquid chromatography (HPLC) are commonly employed to analyze the complex mixture of chemicals in e-liquids, including nicotine, flavoring compounds, and potentially harmful additives [24,25]. On the other hand, elemental analysis is conducted using spectroanalytical techniques, such as inductively coupled plasma (ICP), along with various atomic absorption, emission, and fluorescence spectroscopy methods.

Analytical methods based on these techniques allow researchers to quantify toxicants and evaluate their potential health risks. The role of analytical chemistry in this context is vital, as it helps to reveal the presence of toxic compounds and their interactions, thus providing essential information for public health assessments and regulatory measures. By exposing the negative effects of these products, analytical chemistry supports efforts to mitigate their impact on health and the environment [26].

This review article aims to summarize the information generated over the past five years regarding e-cigarettes, with a particular focus on the policies and regulations that have emerged for these devices. In addition, a thorough analysis of the compounds that have been identified and quantified to date, as well as their impact on environmental public health implications, will be provided. This article will also cover the sample preparation methods and instrumental analytical techniques used in the literature, providing a comprehensive overview of the analytical methods used to assess the composition of e-cigarette liquids and aerosols. Unlike other reviews, this document will emphasize the emergence of new contaminants, such as nitrosamines, that have recently been detected in e-cigarettes and pose potential health risks. Furthermore, special attention will be given to the analysis of metals in these devices, highlighting recent advances in techniques that can improve detection sensitivity and selectivity for trace elements, further contributing to our understanding of the safety and chemical composition of e-cigarettes.

## 2. Materials and Methods

For this review, we carried out an exploratory search using Web of Science (https://www.webofscience.com, accessed on 2 January 2025) (Clarivate Analytics), a multidisciplinary platform whose Core Collection consists of ten indexes covering thousands of academic journals, books, conference proceedings, and collections. Additionally, we consulted ScienceDirect (https://www.sciencedirect.com, accessed on 2 January 2025) (Elsevier), which provides access to a vast collection of peer-reviewed scientific literature, and PubMed (https://pubmed.ncbi.nlm.nih.gov, accessed on 2 January 2025), the database of the National Center for Biotechnology Information (NCBI) of the United States of America, comprising approximately 30 million citations of Medline’s biomedical literature, life science journals, and online books.

The search was completed in January 2025 using the terms “‘electronic cigarette’ and ‘chemical analysis’”, and included publications dating from January 2011 to December 2024. The Boolean operator “and” between the terms was used to refine the search scope, ensuring that results were restricted to the title, abstract, and keywords for a more targeted and effective outcome. Eight researchers conducted the literature search and data collection. Each researcher independently reviewed the literature within assigned time frames, screening titles and abstracts based on predefined inclusion and exclusion criteria. All selected articles were reviewed collectively by the researchers. No automated tools were used in the screening process. The following were excluded: articles not in English, letters to editors, review articles, and studies that did not include any experimental or analytical assessments of e-cigarette aerosols or liquids. In total, more than 140 scientific and review articles were considered to develop this document.

To ensure a structured and comprehensive analysis, the selected articles were classified according to the main topics covered in this review: impacts on health and the environment, sample preparation, gas analysis, liquid analysis, and identified or quantified toxic compounds in e-liquids and aerosols of electronic cigarettes. This categorization facilitated the systematic integration of findings, allowing for a clearer interpretation of the chemical composition of e-cigarette emissions, the methodologies used for their analysis, and their potential risks.

## 3. Inspection and Regulations

Regulations concerning e-cigarettes are subject to ongoing evolution as the scientific understanding of their health effects progresses. Regulatory approaches range from stringent controls to more lenient frameworks, reflecting varying public health priorities across different jurisdictions. In the United States, the FDA has classified e-cigarettes as tobacco products since 2016. This regulatory framework encompasses oversight of manufacturing practices, product labeling, and sales, particularly targeting the prevention of sales to minors [27]. Additionally, manufacturers are mandated to secure premarket authorization for new products. Within the European Union (EU), e-cigarettes are regulated under the Tobacco Products Directive (TPD) [28]. This directive imposes specific limits on nicotine concentration, establishes comprehensive labeling requirements, mandates health warnings, and restricts advertising. It also stipulates that e-liquid containers must not exceed 10 mL and that tanks must be limited to 2 mL in capacity.

In the United Kingdom, the Medicines and Healthcare Products Regulatory Agency (MHRA) oversees e-cigarettes as consumer products rather than medicinal products [29]. The regulations restrict nicotine concentration and packaging sizes. Although the UK adheres to the TPD regulations, it adopts a more flexible approach to vaping, considering it a potentially less harmful alternative to smoking. In Australia, e-cigarettes that contain nicotine are regulated as medicines and require a prescription for legal sale. The Therapeutic Goods Administration regulates these products, imposing stringent controls on their importation and sale without a prescription [30]. In Canada, e-cigarettes are regulated under the Vaping Products Regulations and the Tobacco and Vaping Products Act [31]. These regulations include specific restrictions on nicotine content, labeling, and advertising, with a particular emphasis on preventing access by minors.

In China, which is the largest producer of e-cigarettes, the regulatory environment is less stringent compared to other countries. However, there are ongoing efforts to implement stricter regulations aimed at improving quality control and marketing practices [32]. In Latin America, regulatory approaches to e-cigarettes vary considerably. For example, Brazil has implemented a ban on e-cigarette sales due to health concerns, as outlined by the National Health Surveillance Agency [33]. In Mexico, the circulation and commercialization of Electronic Nicotine Delivery Systems, Nicotine-Free Similar Systems, Alternative Nicotine Consumption Systems, e-cigarettes, and similar vaporizer devices are prohibited by presidential decree [34]. This prohibition also extends to any solutions and mixtures used in such systems, regardless of their origin.

## 4. Bibliometric Analysis

The entire revolution of electronic cigarettes began with the patent of modern electronic cigarettes, which were patented in 2004 by Hon Lik in China [35]. This innovation marked the start of a new era in the tobacco industry and paved the way for the development of a wide range of vaping products. Early research articles on e-cigarettes appeared around 2009–2011, focusing on various aspects such as device efficiency, nicotine content, and health implications [36,37,38,39]. Initial research often concentrated on the potential benefits of e-cigarettes as smoking cessation tools and their chemical composition [40].

**Figure 1:**
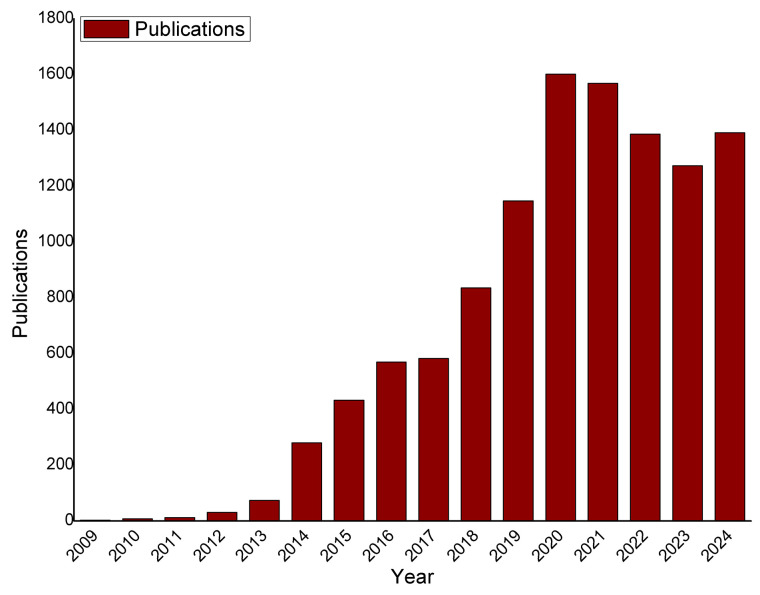
Articles published since the emergence of e-cigarettes. Data taken from the web of science database using the keyword “e-cigarettes”.

A bibliometric analysis of e-cigarettes, conducted using the Web of Science™ database, reveals a broad spectrum of research across multiple fields (Figure 1). In the health domain, studies have extensively examined the health effects of e-cigarettes compared to traditional cigarettes [41]. Research has delved into the exposure to potentially harmful substances, nicotine addiction, and the long-term impacts on respiratory and cardiovascular systems [21,42,43,44]. Systematic reviews and clinical trials contribute to understanding both the risks and potential benefits of e-cigarette use.

Environmental research has highlighted concerns regarding the ecological footprint of e-cigarettes. Studies have investigated the environmental impact of disposable cartridges and batteries, as well as the release of chemical substances into the atmosphere [45]. The management of waste and recycling practices for e-cigarette components are also critical areas of focus.

The social impact of e-cigarettes is another significant research area. Scholars have explored how e-cigarettes influence smoking behaviors, their appeal to younger populations, and the implications for public health policies [46,47]. Surveys and behavioral studies provide insights into the shifting perceptions of smoking risks and the effectiveness of e-cigarettes as smoking cessation aids.

In the realm of chemical analysis, research has scrutinized the composition of e-liquid and vapor produced by e-cigarettes. Analytical studies have identified and quantified various chemical compounds, including nicotine, propylene glycol (PG), and flavoring agents, as well as potential harmful by-products formed during vaporization [48,49]. This chemical scrutiny is essential for assessing the safety and potential risks associated with e-cigarette use.

## 5. Impacts on Health and the Environment

Electronic cigarettes have rapidly become popular and accessible, creating a legion of users, particularly among younger individuals. However, their impact from a clinical and public health perspective remains uncertain [50]. One of the reasons presented for the use of electronic cigarettes is smoking cessation; however, despite being promoted for this purpose, they have not demonstrated effectiveness in tobacco cessation [51,52]. Compared to other therapeutic approaches for quitting smoking, the lack of evidence regarding the safety of electronic cigarettes, combined with the risk of dependence, limits any conclusions regarding the overall balance of benefit and harm associated with the use of electronic cigarettes for smoking cessation [53].

Manufacturers seek to attract and capture users by appealing to various product characteristics, from flavor and aroma to eye-catching advertisements. Some reported reasons for the use of electronic cigarettes among adolescents and young adults include their flavor, discretion, easy accessibility, desire to experiment, perceptions of being safer, and advertising, as well as marketing directly aimed at young people [54].

The liquids heated in electronic cigarettes generate vapors containing substances present in the liquid or formed through the thermal decomposition of the liquid’s ingredients. Some substances are already known for their capacity to produce negative impacts on the human body, such as nicotine, which is commonly found in electronic cigarettes, despite the existence of products marketed as “nicotine-free” [55]. Additionally, other toxic compounds have been identified, including formaldehyde, acetaldehyde, acrolein, and benzene, which are by-products of thermal degradation. Potentially toxic elements, such as lead, cadmium, and nickel, have also been detected [56,57].

The biological effects of nicotine are diverse and include negative impacts on the cardiovascular system, as well as dependence caused by interaction with nicotinic acetylcholine receptors, which initiates the release of neurotransmitters such as dopamine, norepinephrine, acetylcholine, serotonin, Gamma-aminobutyric acid (GABA), glutamate, and endorphins, subsequently inducing sensations of pleasure, stimulation, and mood modulation. The release of catecholamines stimulated by nicotine in the sympathetic nervous system activates β-adrenergic receptors in the heart, resulting in increased heart rate, cardiac contractility, and workload [58,59].

PG and glycerol, used as hygroscopic carriers, have also been shown to induce cardiopulmonary toxicity in animal studies and in vitro [55]. PG has been associated with symptoms such as those of upper respiratory tract infections. Exposure to glycerol is linked to irritation of the eyes, lungs, and esophagus, while its thermal decomposition produces acrolein, a potent irritant for the skin, eyes, and nose, as well as a potential carcinogen. Furthermore, both glycerol and PG exhibit the formation of acetals, indicating the generation of formaldehyde and acetaldehyde—highly toxic compounds that can lead to numerous diseases [60,61]. Similarly, flavor aldehydes such as benzaldehyde, cinnamaldehyde, citral, ethylvanillin, and vanillin react with PG in e-liquids, forming over 40% acetals, which are also present in commercial products [62]. Vaping transfers 50–80% of these acetals to the aerosol, where they remain stable (half-lives > 36 h). Their ability to activate transient receptor potential vanilloid 1 (TRPV1) and ankyrin 1 (TRPA1) suggests potential respiratory effects.

Due to the wide variety of e-liquid flavors available, a classification system has been proposed to categorize them into 13 main groups: tobacco, menthol/mint, nuts, spices, coffee/tea, alcohol, other beverages, fruit, dessert, candy, other sweets, other flavors, and unflavored [63]. The fruit category is further divided into berries, citrus, tropical, and other fruits, following classifications used in flavor wheels for wine, whiskey, coffee, and chocolate.

Spice-flavored flavoring additives have been reported as hazardous to lung health. Cinnamon-flavored cinnamaldehyde has been identified as one of the main constituents capable of damaging immune cells in the lungs, compromising macrophage phagocytosis even in the absence of nicotine. Additionally, other flavors have been associated with increased tumor necrosis factor, interleukin levels, and oxidative stress linked to DNA alterations [54,64]. A recent review article highlighted that while many e-cigarette ingredients, including flavorants and solvents, are Generally Recognized as Safe (GRAS) for food use, their inhalation toxicity remains poorly understood [65]. The lack of inhalation data complicates both the regulation and public perception of the risks associated with e-cigarettes.

In 2019, in the United States, several case reports linked the use of electronic cigarettes to a respiratory syndrome. Cases reported in Illinois, Wisconsin, and Utah, along with a report presenting images of pulmonary changes [66,67,68], described a syndrome where patients exhibited symptoms such as shortness of breath, chest pain, cough, fever, malaise, nausea, vomiting, and abdominal pain. Imaging studies revealed pulmonary lesions, and these patients reported using electronic cigarettes [50].

The syndrome was named e-cigarette or vaping product use-associated lung injury (EVALI) by the U.S. Centers for Disease Control and Prevention (CDC). As of February 2020, 2807 cases had been reported to CDC, with 68 confirmed deaths. Studies linked vitamin E acetate, used as an additive in electronic cigarettes containing THC and cannabidiol (CBD), to the EVALI outbreak, as it was found in bronchoalveolar lavage (BAL) fluid samples from patients with EVALI. Moreover, a significant majority of diagnosed patients reported using products containing THC and CBD [69].

During 2020, due to the COVID-19 pandemic, reports of EVALI cases declined, even though cases continued to occur. Efforts were focused on the rapid progression of the pandemic, presenting challenges for the diagnosis, treatment, and tracking of EVALI incidence. Recent research indicated that EVALI cases are still occurring, and vitamin E acetate remains the likely causative agent of acute lung injury in patients with EVALI [70,71].

The long-term effects of exposure to electronic cigarettes remain uncertain concerning their impact on users. Although pulmonary injury associated with electronic cigarette use is already recognized and vitamin E acetate is identified as a potential causative agent, the pathophysiological mechanisms of the disease have not yet been described, and it is not possible to definitively ascertain how the condition occurs. Regarding smoking cessation, evidence of the effectiveness of electronic cigarettes for this purpose has not been conclusive. There is evidence suggesting that products containing nicotine may play a role in reducing risk for active smokers; however, they may also encourage smoking among non-smokers [72].

These uncertainties are further highlighted by several clinically reported cases suggesting a possible association between e-cigarette use and neoplasm development. For example, a 66-year-old man was diagnosed with basaloid squamous cell carcinoma of the oral cavity after using e-cigarettes 20 times a day for 13 years [73]. Similarly, a 59-year-old man developed basaloid squamous cell carcinoma after 13 years of daily e-cigarette use and presented with a non-healing ulcer on the lower lip [73]. A 19-year-old male was diagnosed with poorly differentiated invasive squamous cell carcinoma of the tongue after four years of daily e-cigarette use [74]. Another case involved a 22-year-old man who developed high-grade conjunctival intraepithelial neoplasia after five years of e-cigarette use [75]. Finally, a 33-year-old man with a history of conventional cigarette smoking was diagnosed with thoracic NUT midline carcinoma after one year of e-cigarette use [76]. These cases highlight the potential risks associated with prolonged e-cigarette use.

**Figure 2:**
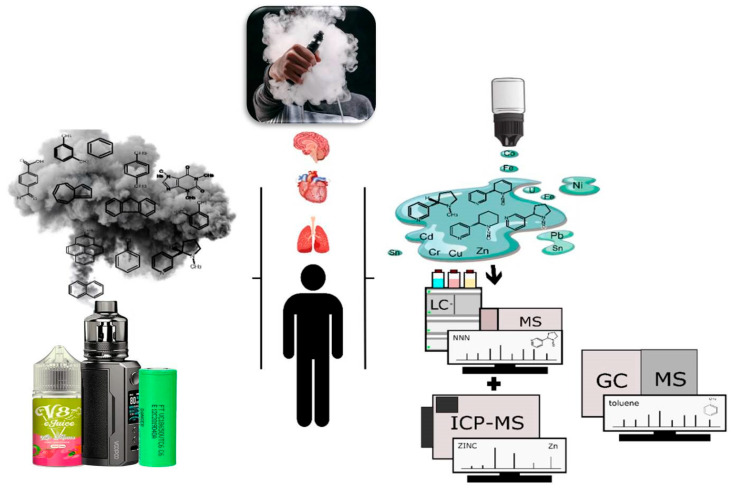
The relationships between sample preparation, analytical techniques, and the risks associated with e-cigarettes.

The eye-catching appearance and marketing strategies that suggest safety contribute to the general population’s normalization of electronic cigarette use. The addition of substances that mimic the flavor and aroma of foods or fruits makes the vapor more palatable, and the appeal to young adults has led to the popularization and prevalence of electronic cigarette use, rendering them a public health threat overall. Figure 2 summarizes the focus of this manuscript by highlighting the interrelationships between sample preparation, analytical techniques, and the risks associated with the chemical species (inorganic and organic) present in e-cigarettes, discussed in the following subsections.

## 6. Sample Preparation and Analysis

Sample preparation for e-liquids can be accomplished using various methods, each with its specific advantages. Headspace sampling is a simple and environmentally friendly technique that allows for the pre-separation of less volatile components such as PG, GLY, and nicotine, facilitating the identification and quantification of volatile compounds like terpenes and cooling agents in e-liquid samples [77]. This method avoids the interference of non-volatile compounds and enables rapid and efficient GC analysis, bypassing complex preparations.

The “dilute-and-shoot” (DnS) approach is commonly used in the characterization of e-liquids due to the high miscibility of the main components (PG and GLY) with frequently used solvents. This method generally involves diluting the e-liquid with an extraction solution compatible with the analytical equipment. Although dilution reduces sensitivity for trace ingredients and contaminants, it is favored for its simplicity and lower solvent usage. In some cases, additional techniques such as solid-phase extraction (SPE) are employed to clean up and concentrate the e-liquid diluents, which may seem contrary to the DnS approach but is useful for analyzing non-traditional components.

Liquid–liquid extraction (LLE) and solid-phase microextraction (SPME) are other employed methods. LLE, which uses immiscible organic solvents to separate analytes, can partially address matrix effects, though it is not always fully effective [78]. SPME, on the other hand, is particularly suitable for volatile compounds and has been used to detect PAHs and flavoring compounds in e-liquids [79]. Additionally, alternative techniques such as ultrasound-assisted solvent extraction with polypropylene membrane bags have been explored for determining trace levels of TSNAs in e-liquids, showcasing the variety of approaches available for analyzing these products [80].

In addition to these methods, the preparation of samples from e-cigarette aerosols involves specific considerations. The conditions for generating and capturing e-cigarette aerosols can vary significantly due to factors such as the diverse characteristics of e-cigarette products, the lack of standardized puffing regimens, limited access to commercial puffing machines, and the need for different trapping methods depending on whether compounds are in the liquid or gas phase of the aerosols [81]. As a result, these variations highlight the importance of adapting sample preparation techniques to the specific requirements of aerosol analysis. In the following sections, a particular emphasis will be placed on the state of the sample, whether gaseous or liquid, to address the most suitable preparation technique for each scenario.

### 6.1. Gas Analysis

For the aerosol analysis of e-cigarettes, headspace sampling is the most widely used technique in the literature. Headspace sampling is a method used in analytical chemistry to analyze the volatile components of a sample. In this technique, the sample is placed in a sealed vial, and the gas phase (headspace) above the sample is analyzed. This allows for the detection and quantification of volatile compounds without direct contact with the sample. It is commonly used in industries such as pharmaceuticals, food and beverages, and environmental testing. Another technique that has been utilized is SPME. SPME is an analytical method used for sampling and analyzing volatile and semi-volatile compounds from various matrices. It involves a coated fiber that adsorbs analytes from a sample, which are then desorbed and analyzed using techniques such as gas chromatography. Table 1 summarizes the studies that have utilized these techniques.

SPME and static headspace extraction techniques are employed in conjunction with gas chromatography coupled with mass spectrometry. This combination facilitates the analysis of compounds with volatile and semi-volatile characteristics or low molecular weight. Among the compounds identified or quantified in e-cigarettes using these methodologies are primarily aldehydes, ketones, alcohols, ethers, halogenated compounds, aromatics, hydrocarbons, and terpenes. Table 1 summarizes some of the most relevant studies in this area and provides an organized overview of the major compounds detected in e-cigarettes.

Despite its widespread use in the analysis of e-cigarette aerosols, GC-MS presents several limitations that must be considered. One of the main challenges is the need for derivatization when analyzing highly polar or thermally labile compounds, which increases the complexity of sample preparation and can introduce variability in quantification. In addition, the complex composition of the aerosol matrix can lead to matrix effects, co-elution, and potential signal suppression, affecting both qualitative and quantitative accuracy. While tandem mass spectrometry (GC-MS/MS) improves selectivity and sensitivity, it remains limited by the availability of reference spectra and the potential for ambiguous fragmentation patterns. Similarly, comprehensive two-dimensional gas chromatography (GC×GC-MS) improves separation efficiency and allows for more detailed chemical characterization, but its application is limited by longer analysis times, increased data complexity, and the need for advanced computational tools for data processing. These challenges highlight the need for complementary techniques to overcome the inherent limitations of gas chromatography-based approaches in e-cigarette aerosol analysis.

### 6.2. Liquid Analysis

The analysis of e-liquids using liquid chromatography has gained significant relevance in studies on the components of liquids used in electronic cigarettes. Recent research has employed various chromatographic techniques, such as liquid chromatography coupled with tandem mass spectrometry (LC–MS/MS), liquid chromatography coupled with high-resolution accurate mass spectrometry (LC-HRAM-MS), liquid chromatography coupled with ultraviolet detection (LC-UV), and ultra-performance liquid chromatography coupled with quadrupole time-of-flight high-resolution mass spectrometry (UPLC-QTOF-HRMS), for the identification and quantification of key compounds in these products, such as cannabinoids, tobacco-specific nitrosamines, flavoring additives, and nicotine. These techniques have also been used to detect contaminants and substances of abuse, such as illegal drugs and cannabinoids in illicit e-liquids, increasing interest in their application both in public health and forensic safety. Table 2 summarizes some of the most relevant studies in this field.

Although LC-MS is a widely used technique for the analysis of e-liquids in e-cigarettes, several limitations must be considered. One of the most critical challenges is ion suppression, where coeluting components in the complex e-liquid matrix interfere with the ionization efficiency of target analytes, leading to signal reduction and compromised quantification. This effect is particularly pronounced in electrospray ionization (ESI), where charge competition between analytes and excipients such as humectants, nicotine, and flavorings can alter detection sensitivity. In addition, the choice of chromatographic conditions, including mobile-phase composition and stationary-phase properties, plays a critical role in separation efficiency and can lead to co-elution, making compound identification more challenging. While tandem mass spectrometry (LC-MS/MS) improves selectivity and sensitivity through multiple reaction monitoring (MRM), it remains susceptible to ion suppression and matrix effects, particularly in highly concentrated or complex samples. Moreover, differences in ionization efficiency between structurally different compounds can introduce bias in comparative quantification. These limitations highlight the need for careful method optimization, the use of stable isotope-labeled internal standards, and rigorous validation protocols to ensure the accurate and reproducible analysis of e-liquid constituents.

## 7. Identified or Quantified Toxic Compounds in E-Liquids and Aerosols of Electronic Cigarettes

Electronic cigarettes, while marketed as safer alternatives to traditional tobacco, can expose users to various toxic compounds. Metals like lead and nickel, PAHs, nitrosamines, and nicotine have been detected in e-liquids and aerosols. This section examines the presence and implications of these toxicants, highlighting the importance of monitoring and regulation.

### 7.1. Nicotine

Nicotine is an alkaloid extracted from the leaves of the tobacco plant; it is a weak base and is soluble in water. Nicotine has a pKa of 8.0, at which pH 50% of it exists in its free base form and 50% is ionized. When the vapor of an electronic cigarette is inhaled, aerosols containing nicotine are generated and transported to the lungs [104].

With the increasing popularity of e-cigarettes, the development of analytical methods for quantifying the key components in e-liquids, such as nicotine, has garnered attention, contributing to the assessment of their associated health risks. Recent research, particularly from the last five years, reveals a variety of methods used to identify and quantify nicotine in electronic cigarettes, from the e-liquid to the vapor generated by the device.

Lee et al. [105] developed a method using gas chromatography capable of identifying and quantifying nicotine in samples. The method was applied to e-liquids claiming to be nicotine-free, confirming the absence of nicotine in the samples. Kubica [106] developed and applied a method using liquid chromatography, with results aligning with the nicotine content stated on the labels of e-liquids, which ranged from 0 mg/mL for nicotine-free products to 3.00–12.00 mg/mL for those claiming to contain nicotine. Dai et al. [107] quantified nicotine in both e-liquids and aerosols generated by vaporizing these e-liquids using electronic cigarettes. The method developed and validated by the authors demonstrated consistency between the declared nicotine levels and those determined, ranging from 6.76 to 16.3 mg/g in e-liquids. In aerosols, the concentrations ranged from 5.7 to 14.7 mg/g, which were lower than those in liquid samples.

Alhusban and Ata [108] quantified nicotine in their samples, concluding that the actual values were lower than those declared on the e-liquid labels, with concentrations ranging from 0 to 25.81 mg/mL. Barhdadi et al. [109] developed, validated, and applied their method to quantify nicotine impurities and other alkaloids, such as cotinine and anabasine. Four samples were labeled as nicotine-free, and two of these contained trace amounts of nicotine. In the other six samples claiming to contain nicotine, the method proved suitable for identifying and quantifying nicotine. Similarly, Krüsemann et al. [49] identified discrepancies in nicotine labeling in e-liquids, detecting nicotine in 5% of the products marketed as nicotine-free and failing to detect it in some labeled as containing 6 mg/mL.

These studies collectively underscore the importance of accurate labeling and the need for robust analytical methods to ensure the safety and transparency of e-cigarette products. The discrepancies between labeled and measured nicotine concentrations not only raise concerns about consumer trust, but also highlight potential health risks associated with unintended nicotine exposure. Moreover, the variability in nicotine levels in aerosols compared to e-liquids demonstrates the complex dynamics of nicotine delivery in e-cigarette systems.

### 7.2. Polycyclic Aromatic Hydrocarbons (PAHs)

PAHs are organic compounds formed by two or more fused aromatic rings. Due to their stable chemical structure, PAHs do not easily decompose in the environment, which contributes to their persistence and potential for bioaccumulation. These compounds are well known for their adverse health effects, particularly their carcinogenic nature when ingested or inhaled [110]. The United States Environmental Protection Agency (EPA) has classified 16 PAHs as priority pollutants due to their high carcinogenicity [111]. Among these are genotoxic compounds such as benzo[a]anthracene (B[a]A), chrysene (CHRY), benzo[b]fluoranthene (B[b]F), benzo[k]fluoranthene (B[k]F), benzo[a]pyrene (B[a]P), dibenz[a,h]anthracene (D[ah]A), and benzo[g,h,i]perylene (B[ghi]P), due to their particularly harmful effects on the body. The list also includes compounds such as naphthalene (NAP), acenaphthylene (ACY), acenaphthene (ACE), fluorene (FLU), phenanthrene (PHEN), anthracene (ANTH), fluoranthene (FLTH), pyrene (PYR), and indeno [1,2,3-c,d]pyrene (IND).

The detection and quantification of PAHs in electronic cigarettes are highly relevant due to the toxicity and carcinogenic potential of these compounds. For the quantification of PAHs in e-liquids and aerosols from electronic cigarettes, HPLC and GC are commonly employed, often coupled with mass spectrometry or fluorescence detectors. PAHs are primarily generated during the combustion or heating of organic components. Although electronic cigarettes do not produce combustion, certain heating conditions within these devices can lead to PAH formation. The generation and release of PAHs in electronic cigarette aerosols depend on various factors, notably the heating temperature and the type of device used. While some studies have shown that heating temperatures above 300 °C generate PAHs [112,113], other reports indicate that low temperatures can also lead to the formation of compounds such as phenanthrene and anthracene [114,115].

In the literature, no quantitative studies on these compounds in aerosols have been reported thus far. However, qualitative analyses conducted to date have confirmed the presence of certain PAHs. NAP, along with its mono- and polysubstituted derivatives, is among the compounds identified [77]. Furthermore, in a quantitative analysis of e-liquids, PAHs such as ACE, ACY, B[a]P, B[b]F, B[ghi]P, CHRY, FLTH, FLU, NAP, and PHEN were detected at concentrations of ≤4.1 pg/mL per puff [116].

### 7.3. Nitrosamines (TSNAs)

For decades, the presence of nitrosamines in tobacco used for traditional cigarettes has been extensively studied, with significant focus on the toxicological effects of TSNAs and meta-analyses [117,118,119]. However, research on nitrosamines in e-liquids, particularly those atomized by modern nicotine delivery systems like e-cigarettes, remains in its early stages. Although over 300 nitrosamines are recognized, many of which are potentially carcinogenic [120], to the best of our knowledge, no significant studies have been conducted on the formation of nitrosamines other than TSNAs through e-cigarettes in the last five years. Thus, the potential formation of non-tobacco-related nitrosamines in e-cigarettes remains an underexplored topic.

**Figure 3:**
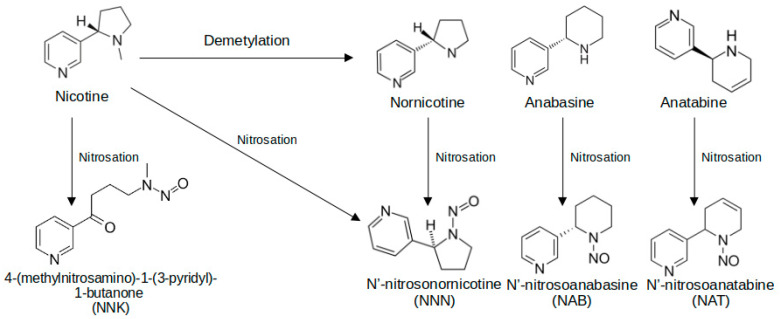
Mechanism of TSNA formation during tobacco curing and e-cigarette operation.

TSNAs are primarily formed during certain types of curing processes of tobacco leaves, including processing stages such as fermentation and aging, as well as long-term storage [121]. The widely accepted hypothesis for the formation of these toxicants centers on the microbial reduction of nitrate (found primarily in fertilizers) to nitrite and other nitrogen oxides (NOx), which act as oxidizing agents, and then, these NOx species participate in nitrosation reactions with tobacco alkaloids [122], including nicotine, nornicotine, anatabine, and anabasine, leading to the formation of 4-(methylnitrosamino)-1-(3-pyridyl)-1-butanone (NNK), N′-nitrosonornicotine (NNN), N′-nitrosoanatabine (NAT), and N′-nitrosoanabasine (NAB), respectively (Figure 3). Just as TSNAs can be formed during the harvesting stages of tobacco leaves, they can also be generated during the operation of e-cigarettes. In a recent study, Jin et al. [123] demonstrated that TSNAs can be formed in situ, with their content varying significantly depending on factors such as temperature, nitrite concentration, nicotine concentration, and other alkaloid impurities.

These four substances have been under investigation by the WHO International Agency for Research on Cancer since 1985 [124,125], as they are the predominant TSNAs found in traditional cigarette smoke [126]. They continue to be the central focus of contemporary research on TSNAs in e-liquids and the aerosols produced during the vaping process.

Another related substance is 4-(methylnitrosamino)-1-(3-pyridyl)-1-butanol (NNAL), a reduced derivative of NNK that is frequently recognized as a metabolite and thus serves as a biomarker for NNK contamination. NNK is a recognized carcinogenic agent responsible for certain types of cancer in the oral cavity, as well as lung cancer, and is listed by the FDA as a harmful constituent in tobacco [127]. For instance, Matínez-Sánchez et al. indicate a correlation between NNAL levels in the urine of both e-cigarette users and individuals exposed to secondhand aerosol. In a past study, this same nitrosamine has been detected in the saliva of people exposed to secondhand smoke [128]. While levels of this substance are low in non-smokers, they increase with the intensity and duration of exposure [129,130] and vary according to the usage status of nicotine-based products. In a study conducted between 2016 and 2018, which analyzed the urine of 2845 individuals, NNAL levels were found to vary significantly between ex-smokers (1.974 ± 0.091 pg/mL), habitual e-cigarette smokers (14.349 ± 5.218 pg/mL), and dual users of both e-cigarettes and traditional cigarettes (89.002 ± 11.444 pg/mL) [131].

TSNAs in e-liquids and aerosols have been determined using a variety of combinations of chromatographic analytical systems. As a relatively recent topic, these methods are still considered experimental and innovative, lacking established analytical protocols and standardization, although Lee et al. [132] suggest comparing the established method with international standards such as those set by the WHO [133] for nitrosamines in traditional cigarettes [134]. Wang et al. [99] established a semi-quantitative method based on UPLC-QTOF-HRMS. On the other hand, Kubica [80] utilized LC–MS/MS and employed gas chromatography with flame ionization detection (GC-FID) and gas chromatography with thermal conductivity detection (GC-TCD). In general, there are limited studies that evaluate the emissions of TSNAs released using e-cigarettes with confidence, and jointly, only a few trends can be identified.

Previous studies have indicated a strong correlation between nicotine concentration and TSNA content [129,135]. However, Lee et al. [132] reported that none of the four TSNAs (NNK, NNN, NAT, and NAB) were detected in commercial e-liquids, possibly due to the low nicotine concentration observed in the sampled e-liquids (8.18 ng/g). In a cross-sectional study, Smith et al. [136] evaluated a large sample of individuals, comprising 456 people across three countries: the United States, the United Kingdom, and Poland. By comparing biomarkers in urine samples from four groups using CDC methodology (e-cigarette users, traditional cigarette users, users of both e-cigarettes and traditional cigarettes, and a control group of non-users), they observed that the profile of nicotine toxicants, including TSNAs and their metabolites, was consistent across countries. Additionally, e-cigarette users exhibited higher levels of TSNAs and their metabolites compared to traditional cigarette smokers, which contrasts with previous studies suggesting that exposure to nicotine and its toxicants from e-cigarettes is lower or comparable to traditional cigarettes [137,138], as well as with the misconception that e-cigarettes represent a “healthier” alternative.

Newer e-cigarette devices can be loaded with e-liquids containing higher concentrations of nicotine and atomize significantly more nicotine than traditional cigarettes, thus exposing users to higher levels of TSNAs compared to other groups [136]. Additionally, older surveys indicate that e-cigarette users inhale more than four times the amount of nicotine-containing vapor compared to traditional cigarette smokers [139,140]. This underscores the urgent need for more comprehensive toxicological studies to assess the health impacts of TSNAs emitted during the vaping process on both smokers and individuals exposed to secondhand aerosols. Frequency of use is a factor often overlooked in modern studies, and new forms of nicotine, such as nicotine salts, have been shown to provide greater satisfaction compared to nicotine in its native form [141,142]. This has been linked to increased dependence and a heightened desire for higher doses of this substance.

### 7.4. Potentially Toxic Metals (PTMs)

The literature reveals a clear trend in the development of quantitative methods for potentially toxic metal (PTM) determination, predominantly centered on the inductively coupled plasma mass spectrometry (ICP-MS) technique. However, there are also a few reports of methods developed using more cost-effective techniques, such as total reflection X-ray fluorescence (TXRF) and inductively coupled plasma optical emission spectrometry (ICP OES) (Table 3). Studies typically focus on six key elements—Cd, Cr, Cu, Ni, Pb, and Zn—with the most frequently analyzed matrices being both the e-liquid and the aerosol produced during the vaping process. To the best of our knowledge, most studies emphasize the determination of total metal content in these matrices, with few addressing the development of chemical speciation methods. The concentration ranges found for each metal were as follows: Pb (1.0 to 2560 µg kg^−1^); Cd (0.04 to 141 µg kg^−1^); Ni (0.73 to 61,300 µg kg^−1^); Cr (0.05 to 5330 µg kg^−1^); As (0.11 to 8.3 µg kg^−1^); Al (5.39 to 34.7 µg kg^−1^); Co (510 to 550 µg kg^−1^); Cu (4.0 to 927,000 µg kg^−1^); U (0.04 to 0.06 µg kg^−1^); Mn (0.42 to 96.1 µg kg^−1^); W (0.05 to 0.15 µg kg^−1^); Sn (0.37 to 58,200 µg kg^−1^); Zn (387 to 4540 µg kg^−1^); and Fe (4.44 to 200 µg kg^−1^). Cu, Ni, and Sn exhibit the highest concentration intervals, indicating their potential relevance in assessing exposure risks.

In general, while trace elements are detected in e-liquids, with concentrations varying considerably depending on the manufacturer and batch [153], the literature suggests that the primary source of PTMs found in the vapor inhaled during the vaping process is the physical composition of the e-cigarette itself, with small transfers ranging from 1% to 4.7% directly from the e-liquid to the aerosol [146]. In this context, Kim et al. [154] measured the concentration of eight elements in aerosols produced from an e-liquid candidate for reference material (RM). Initially, all metals had concentrations below the limit of detection (LOD) when using a new e-cigarette. However, after four months of use, with daily cycles of 20 h, Mn (0.001 mg/L) and Pb (0.097 mg/L) were detected in the aerosol. Similarly, Mallampati et al. [155] observed a significant increase in Ni levels after subjecting Cannabis sativa concentrates to the vaping process, which is consistent with the findings of Omaiye et al. [156], who reported Ni and Cr particles in pod-style electronic cigarettes. In addition, Hess et al. [157] observed wide fluctuations in the concentrations of PTMs in other components of electronic cigarettes.

In fact, vape cartridges can vary in shape and physical characteristics during manufacturing. In this context, studies reporting the direct analysis of internal components of vapes using characterization techniques such as scanning electron microscopy (SEM) have provided valuable insights into surface morphology, elemental composition, and degradation [158,159,160]. In general, solder joints and battery connections often contain Pb, Sb, and Sn, as well as alloys such as nichrome, chromel, inconel, elinvar, invar, and kanthal, contributing Al, Cr, Fe, and Ni to the inhaled vapors.

In a more in-depth study comparing e-cigarette devices from different years (generations) and manufacturers, filled with their respective e-liquids, Na et al. [161] found that the date of manufacture, along with design changes, plays a significant role in the level of PTMs detected in the aerosol. In older devices, e-liquids remain in contact with internal components for longer periods, promoting the greater oxidation and leaching of metal particles. Metal levels, particularly Cu and Zn, were found to be higher in older devices, which often used bronze connectors, components that have since been discontinued in more modern products. Furthermore, in modern vape models, such as cartomizers and disposables, the coils are coated with a thin layer of plastic or Teflon^®^ instead of silver-coated copper or nickel/nichrome coils [160]. While this coating may limit the transfer of metals into the aerosol, it can contribute to the release of harmful organic substances due to thermodegradation of these polymers during vaping.

These findings elucidate that the direct contact between e-liquids and other products with the atomization coil and other internal components, combined with the high temperatures reached and the air drawn into the atomization chamber, promote the oxidation of metals and the transfer of coil and electronic component materials into the vapors inhaled by the user. In fact, it was observed that the metal content increased significantly (by a factor of 7 to 631) when the filament heating power was raised from 20 to 40 W [151]. Higher power levels also led to an increase in the average size of particles released from the atomization coil [162]. Additionally, it was demonstrated that the regular surface of the metal filaments deteriorates rapidly, developing critical fissures after approximately 150 puffs [158]. This finding suggests that metal transfer increases with prolonged use.

This dynamic is further intensified by the presence of certain substances in e-liquids, which can undergo thermal conversion into highly oxidizing and/or complexing compounds (e.g., acetaldehyde, pyridine derivatives, quinolines, arylnitriles, and aromatic hydrocarbons, among others). These compounds accelerate the oxidation process and enhance the sequestration of metals from the internal surfaces of e-cigarettes [150]. However, as observed in the study of Gray et al. [144], this process does not appear to be pH-dependent within the acidity range of the tested e-liquids, and Zhao et al. [152] did not observe a clear correlation between nicotine content and the total metal content in the aerosol. Therefore, the relationship between the chemical composition of e-liquids and the PTM content necessitates further investigation.

Considering a standard puff volume of 55 mL [162,163,164], an average e-liquid density of 1.12 g/mL [165], and a consumption of 10 puffs per day, which is the standard amount defined by the World Health Organization [164], It is evident that individuals in this group may be exposed to PTM levels exceeding the maximum limits set by organizations such as the International Council for Harmonization of Technical Requirements for Pharmaceuticals for Human Use [166], Agency for Toxic Substances Disease Registry [167], and National Institute for Occupational Safety and Health [168] (Table 4).

Although ICP-MS is a highly sensitive and versatile technique for the elemental analysis of e-liquids, several limitations must be considered. One of the primary challenges is plasma tolerance, as samples with a high matrix content (>0.3%) can cause ionization suppression, signal drift, or even the clogging of interface cones, affecting long-term stability and reproducibility. Additionally, spectral interferences arising from the sample matrix can affect accuracy, as they are often variable and unpredictable, depending on the elemental composition of the e-liquid. These interferences can originate from polyatomic species, doubly charged ions, or oxide and hydroxide formations, complicating the identification and quantification of trace elements. Another significant limitation is the restricted linear dynamic range, particularly when analyzing elements present at both trace and major levels. High-sensitivity configurations optimized for ultra-trace detection can lead to signal saturation for major elements, requiring careful method optimization, dilution strategies, or the use of collision/reaction cell technology to minimize such effects. These factors underscore the need for rigorous calibration protocols, matrix-matching strategies, and proper instrument maintenance to ensure the reliable and accurate elemental analysis of e-liquids.

This raises serious health concerns regarding the potential toxicological impacts of PTMs present in vaping products, emphasizing the need for stricter regulatory measures and the continuous monitoring of e-cigarette components. Also, Abdelghani et al. [143] revealed that the accumulation of metals such as Ni, Cr, Cu, and Pb in the e-liquid residue left on wick/cotton after the vaping process is significantly higher—ranging from 2 to 37 times higher—than those typically found in e-liquids prior to use, and due to their known bioaccumulative properties this may lead to long-term deleterious effects. For example, Cr, Pb, and Ni are known carcinogens [169,170,171,172,173], and long-term exposure to Pb can lead to cardiovascular, neuronal, and kidney diseases [174], in addition to being possibly related to the emergence of lung fibrosis [175]. Furthermore, the presence of Pb, which primarily arises from solder joints, is prohibited in China [159], and its detection in vape devices indicates non-compliance with the country’s manufacturing regulations. In other words, vapes not only represent a public health concern, but many manufacturers engage in practices that border on illegality to minimize production costs. This approach exposes workers, users, and the environment to pollutants that are internationally banned.

## 8. Conclusions and Perspectives

Although several review articles have addressed aspects of e-cigarette composition and health risks [176,177,178], the rapid growth of scientific interest in this area, coupled with the continuous emergence of new data and insights, makes it essential to periodically update the literature.

The analysis of e-cigarettes has advanced significantly with the development of sophisticated techniques such as gas chromatography and mass spectrometry, which allow for detailed identification of the volatile and semi-volatile compounds present in these products. However, research on the long-term effects of inhaling e-cigarette vapor remains insufficient. While e-cigarettes are considered less harmful than traditional cigarettes, some studies have detected potentially toxic compounds in e-cigarette liquids, raising concerns about health risks. Additionally, the increase in e-cigarette use among adolescents raises concerns about nicotine addiction and its potential negative effects on pulmonary and neurological development.

Comparative studies of e-cigarettes and other emerging tobacco products, such as heated tobacco products (HTPs) and nicotine pouches, should be expanded to provide a clearer understanding of their potential health risks. While these products are often marketed as safer alternatives to conventional cigarettes, they could contain the same toxic compounds found in e-cigarettes.

Despite the progress in chromatographic and mass spectrometry techniques, their improvements in sensitivity and resolution may not be as critical in the analysis of e-cigarettes due to the lack of established regulations and legal standards regarding permissible concentrations of substances in these devices. As a result, the demand for ultra-sensitive techniques is less urgent in the context of e-cigarette composition, where regulatory frameworks are still under development.

However, when it comes to analyzing metals and other trace elements, there is considerable potential to explore newer techniques that could provide valuable insights into the safety of e-cigarette products. One promising emerging technology is Laser-Induced Breakdown Spectroscopy (LIBS), which can effectively detect metals and other inorganic elements with minimal sample preparation [179]. LIBS works by generating plasma via a laser, exciting atoms in the sample and emitting characteristic spectra for real-time analysis. Although it has not yet been applied in e-cigarette analysis, LIBS could be useful for detecting harmful metals such as lead, cadmium, and nickel at low concentrations.

In the electrochemical domain, recent advancements in techniques like Electrochemical Impedance Spectroscopy (EIS) and Differential Pulse Anodic Stripping Voltammetry (DPASV) show promise in improving sensitivity and selectivity for detecting trace metals [180]. These methods enable real-time monitoring with high precision, which could enhance safety assessments. Similarly, Square Wave Anodic Stripping Voltammetry (SWASV) [181] and Cyclic Voltammetry (CV) [182] could also be explored for analyzing metals and organic compounds in e-cigarettes.

From a spectroscopic perspective, graphene-based sensors could offer a new approach to detecting volatile organic compounds with high sensitivity. These sensors leverage the unique properties of graphene for rapid detection with low detection limits. Additionally, Two-Dimensional Infrared Spectroscopy (2D-IR) could provide detailed molecular information, making it valuable for identifying chemical species [180].

In terms of environmental impact, the waste generated by e-cigarettes, including plastic cartridges and lithium batteries, presents a significant challenge. Improper management of these wastes can contribute to pollution and have adverse environmental effects. Moreover, the release of certain chemical compounds during e-cigarette use could have negative consequences that are not yet fully understood, highlighting the need for further research on these impacts.

In terms of health, despite knowing the concentrations of heavy metals in aerosols and e-liquids, we still do not have a clear understanding of the concentrations that are entering the body. The analysis of these metals reveals their specific effects on the body, with each metal presenting distinct toxicological concerns. Lead (Pb) increases oxidative stress, causing lipid peroxidation and damage to cell membranes, resulting in cellular damage and neurotoxic effects [183]. Cadmium (Cd) is associated with various cancers (lung, prostate, kidney), renal damage, cardiovascular problems, and chronic pulmonary damage [184]. Nickel (Ni) induces epigenetic alterations that can disrupt the genome, as well as inflammatory and allergic responses [185]. Chromium (Cr) causes DNA damage, cancer, and gastrointestinal issues (burns, ulcers), along with reproductive problems [186]. Additionally, vitamin E acetate, which has been found in some e-liquids, has raised concerns due to its potential role in lung injury. When inhaled, vitamin E acetate may break down into toxic compounds that can lead to severe respiratory issues [70,71].

To fully assess these health risks, it is essential to understand the mechanisms and long-term effects of exposure to these metals and chemicals. Future studies are crucial to measure the concentration of these substances in biological matrices such as blood, urine, and saliva in electronic cigarette users. These analyses will be essential for correlating metal and chemical exposure with clinical and toxicological effects and for establishing safer exposure limits, which could guide future regulatory policies on these products.

Regulatory bodies are in the process of developing and adjusting standards to address concerns related to the safety and quality of e-cigarettes. This includes implementing restrictions on sales and advertising, particularly to protect minors, as well as requiring greater transparency regarding the composition of e-liquids and manufacturing practices. These measures aim to balance the need for innovation in consumer products with the protection of public health and the environment.

The field of e-cigarettes has undergone significant evolution in recent years, reflecting both technological advancements and shifts in public and regulatory perceptions. Initially conceived as a safer alternative to traditional cigarettes, e-cigarettes have gained popularity under the assumption that they provide a smoking experience with reduced exposure to the harmful chemicals found in conventional cigarettes. However, as more studies are conducted, the image of e-cigarettes has become increasingly controversial, revealing health risks associated with their use for both young and old smokers.

The regulation of e-cigarettes varies widely across countries, reflecting different approaches to their use and commercialization. In some regions, e-cigarettes are subject to stringent regulations that restrict their sale and advertising, while in others, their availability is much more liberal.

## Figures and Tables

**Table 1:** Summary of key studies on the analysis of e-liquids and aerosols using gas analysis.

Matrix Analyzed	Number of Compounds	Identified or Quantified Compounds (CAS)	Instrumental Technique	Reference
Blood orange-flavored e-liquid	37	Ethanol (64-17-5), Ethyl acetate (141-78-6), 1-Butanol (71-36-3), 1-Butanol (105-37-3), 3-Methyl-1-butanol (123-51-3), 1,2-Propanediol (57-55-6), Isobutyl acetate (110-19-0), Ethyl butanoate (105-54-4), Butyl acetate (123-86-4), Ethyl 2-methylbutanoate (7452-79-1), 3-Hexen-1-ol (Z) (928-96-1), 1-Hexanol (111-27-3), 3-Methylbutyl acetate (123-92-2), 2-Methylbutyl acetate (624-41-9), Ethyl 4-pentenoate (1968-40-7), Heptanal (111-71-7), (+)-α-Pinene (7785-26-4), 1-Heptanol (111-70-6), (−)-α-Pinene (7785-70-8), 6-Methyl-5-hepten-2-on (110-93-0), β-Myrcene (123-35-3), Ethyl hexanoate (123-66-0), Octanal (1234-13-0), Limonene (138-86-3), Eucalyptol (470-82-6), 3-Methylbutyl butanoate (106-27-4), 1-Octanol (111-87-5), Linalool (78-70-6), Nonanal (124-19-6), Citronellal (106-23-0), Menthon (126-81-8), Menthol (89-78-1), Decanal (112-31-2), Geraniol (106-24-1), Carvone (99-49-0), Undecanal (112-44-7), nicotine (54-11-5)	HS-GCxIMS ^#^ and HS-GC-MS	[82]
146 e-liquids (flavor categories: brown, fruit, hybrid dairy, menthol, mint, none, tobacco, and others)	51	Ethanol (64-17-5), Acetaldehyde (75-07-0), D-Limonene (5989-27-), Isopropyl alcohol (67-63-0), Acetone (67-64-1), 2,3-Butanedione (431-03-8), α-Pinene (80-56-8), 2,3-Pentanedione (600-14-6), Benzene (71-43-2), m,p-Xylene (108-38-3, 106-42-3), Toluene (108-88-3), o-Xylene (95-47-6), 2,3-Hexanedione (3848-24-6), Methylene chloride (75-09-2), Ethylbenzene (100-41-4), Methyl methacrylate (80-62-6), n-Hexane (110-54-3), Styrene (100-42-5), Chloroform (67-66-3), Ethyl acetate (141-78-6), Ethyl butanoate (105-54-4), Ethyl propionate (105-37-3), Ethyl 2-methylbutanoate (7452-79-1), Isoamyl acetate (123-92-2), β-Pinene (127-91-3), 2-Methylbutyl acetate (624-41-9), Isopentyl isovalerate (659-70-1), Isobutyl acetate (110-19-0), p-Cymene (99-87-6), Ethyl 3-methylbutanoate (108-64-5), γ-Terpinene (99-85-4), 1,3-Dioxolane, 2,2,4-trimethyl (1193-11-9), Ethyl hexanoate (123-66-0), Benzaldehyde (100-52-7), Hexyl acetate (142-92-7), Isobutyl isovalerate (589-59-3), Pentyl acetate (628-63-7), Cyclohexanone, 5-methyl-2-(1-methylethyl)-, (2R-cis) (1196-31-2), Camphene (9-92-5), β-Ocimene (13877-91-3), α-Thujene (2867-05-2), 1,1-Diethoxyethane (105-57-7), Terpinolene (586-62-9), 4-Hexen-1-ol, acetate (7223736-6), (Z)-Ocimene (3338-55-4), 2-Heptanone (110-43-0), Butyl isovalerate (109-19-3), Methylcyclopentane (96-37-7), Phellandral (21391-98-0), 1,8-Cineole (470-82-6)	HS-GC-MS	[83]
Flavorless e-liquid and a banana-flavored liquid	50	Ethanol (64-17-5), 2-Methylpropanal (78-84-2), Diacetyl (431-03-8), 2-Butanone (78-93-3), Ethyl acetate (141-78-6), Isobutanol (78-83-1), 2-Methylbutanal (96-17-3), 3-Methylbutanal (590-86-3), 2-Pentanone (107-87-9), Ethyl propanoate (105-37-3), Isoamyl alcohol (123-51-3), Methyl isobutyl ketone (108-10-1), Propylene glycol (57-55-6), Isobutyl acetate (110-19-0), 2,3-Hexanedione(110-13-4), 2-Hexanone (591-78-6), Hexanal (66-25-1), Butyl acetate (123-86-4), Furfural (98-01-1), Ethyl 2-methylbutanoate (7452-79-1), trans-2-Hexenal (1335-39-3), trans-2-Hexenol (928-95-0), 3-Methylbutyl acetate (123-92-2), 2-Methylbutyl acetate (585-07-9), 2-Heptanone (110-43-0), Ethyl pentanoate (539-82-2), Methyl hexanoate (106-70-7), (+)-α-Pinene (80-56-8), Benzaldehyde (100-52-7), 1-Octen-3-one (4312-99-6), 2-Octanone (111-13-7), Octanal (124-13-0), trans-2,4-Heptadienal (4313-03-5), D(+)-Limonene ( 5989-27-5), Isoamyl butanoate (106-27-4), 1-Octanol (111-87-5), Allyl hexanoate (123-68-2), 2-Nonanone (821-55-6), Linalool (78-70-6), Isoamyl isovalerate (659-70-1), L-Menthone (14073-97-3), D-Menthone (89-80-5), D/L-Menthol (89-78-1), 2-Decanone (693-54-9), Decanal (112-31-2), Neral (106-26-3), L-Carvone (6485-40-1), Geranial (106-24-1), trans-Anethole (4180-23-8), and Eugenol (97-53-0).	HS-GCxIMS ^#^ and HS-GC-MS	[84]
129 e-liquids	807	807 individual substances were identified, including nicotine, nicotine impurities, VOC impurities, additives (diacetin), and flavoring substances (incl. synthetic substances and components from essential oils or other herbal extracts)—list of compounds not reported by the authors	HS-GC-MS	[85]
Commercially available e-liquids	10	Menthol (2216-51-5), Menthone (14073-97-3), Geraniol (106-24-1), Linalool (78-70-6), 2,3-Butanedione (431-03-8), 2,3-Pentanedione (600-14-6), 2,3-Hexanedione (3848-24-6), 2,3-Heptanedione (96-04-8), Methyl eugenole (93-15-2), Estragole (140-67-0)	HS-GC-IMS/MS	[86]
Mint-flavored and menthol-flavored e-cigarettes	2	Pulegone (89-82-7), menthol (2216-51-5)	HS-GC-MS	[87]
Flavored e-liquid	2	Diacetyl (431-03-8) and acetylpropionyl (600-14-6)	HS-GC-MS	[88]
Flavored e-liquid	1	Ethanol (64-17-5)	HS-GC-FID	[89]
Flavored e-liquid	26	2-Methylpropanal (78-84-2), Diacetyl (431-03-8), Ethyl acetate (141-78-6), 2-Methylpropanol (78-83-1), 2-Methylbutanal (96-17-3), Isoamyl alcohol (123-51-3), Methyl isobutyl ketone (108-10-1), 2,3-Hexanedione (3848-24-6), Hexanal (66-25-1), Butyl acetate (123-86-4), Furfural (98-01-1), Ethyl 2-methylbutanoate ((E)-2-Hexenal) (6728-26-3), (E)-2-Hexenol (928-95-0), Ethyl pentanoate (539-82-2), Methyl hexanoate (106-70-7), (+)-α-Pinene (7785-70-8), Benzaldehyde (100-52-7), 1-Octen-3-one (4312-99-6), Octanal (124-13-0), (E,E)-2,4-Heptadienal (05.03.4313), D(+)-Limonene (5989-27-5), Octanol (111-87-5), (821-55-6), Linalool (78-70-6), L/D-Menthone (89-80-5), D/L-Menthol (1490-04-6), Neral/Geranial (5392-40-5)	HS-GCxIMS ^#^ and HS-GC-MS	[90]
Flavored e-liquid (flavor categories: vanilla, butterscotch, tobacco, cinnamon, apple, menthol, and others)	≈1000	1000 individual substances were identified, including carboxylic acids (18), esters (204), aldehydes (73), ketones (131), alcohols (163), ethers (112), halogenateds (44), aromatics (287), hydrocarbons (alkanes, alkenes, alkines) (267), and miscellaneous (56)	HS-SPME-GC × GC-TOF-MS	[77]
Commercially available e-liquids (two brands)	92	92 VOCs were identified, including 31 esters, 18 alcohols, 10 hydrocarbons, 8 carbonyl compounds, 7 acetals, 5 pyrazines, 4 terpenes, 3 miscellaneous, 2 carboxylic acids, 2 lactones, 1 amine, and 1 volatile phenol	HS-SPME-GC–MS	[91]
Breath analysis (e-liquid flavors)	206	Propylene glycol, hydrocarbons (58), chloride compounds (3), alcohols (23), aldehydes (16), ketones (22), acids (7), esters (33), ethers (1), furans and pyrans (13), nitrogen compounds (14), and aromatic hydrocarbons (16)	HS-SPME-GC–MS	[92]
Three lab-formulated e-liquids	1	Methamphetamine (537-46-2)	HS-SPME-GC–MS, SPME-DART-MS^+^	[93]
Cannabis vape oil liquid, vapor and aerosol	206	100 terpenes and natural extracts, 19 cannabinoids, and other potential toxic additives such as vitamin E acetate, polyethylene glycols, and medium-chain triglycerides	HS-SPME-GC–MS	[94]
Two different types of e-cigarettes and different puff durations were evaluated	3	Formaldehyde (50-00-0.), acetaldehyde (75-07-0), and acrolein (107-02-8)	HS-SPME-GC–MS	[57]
225 replacement liquids were purchased from 17 e-cigarette shops	3	Formaldehyde (50-00-0.), acetaldehyde (75-07-0), and acrolein (107-02-8)	HS-SPME-GC–MS	[95]
Electronic cigarette liquids and aerosols	1	Nicotine (65-31-6)	HS-SPME-GC–MS	[96]
Three commercially available e-liquids	1	MDMB-fubinaca (Methyl (*S*)-2-(1-(4-fluorobenzyl)-1*H*-indazole-3-carboxamido)-3,3-dimethylbutanoate) (1971007-93-8)	HS-SPME-GC–MS	[97]
Flavored e-liquid	72	Ethanol (64-17-5) 1,3-Butadiene, 2-methyl (78-79-5) Propanal (123-38-6) Acetone (67-64-1) Dimethyl sulfide (75-18-3) 1,3-Cyclopentadiene (542-92-7) 2-Butene, 2,3-dimethyl- (563-79-1) 1-Propanol (71-23-8) Furan, 3-methyl (930-27-8) Ethyl acetate (141-78-6) Hexane, 2-methyl (591764) Pentane, 2,3-dimethyl (565-59-3) Hexane, 3-methyl (589-34-4) Benzene (71-43-2) Heptane (142-82-5) Acetic acid (64-19-7) Propane, 1-(methylthio)- (3877-15-4) Furan, 2,5-dimethyl (625-86-5) 2-Pentanone (107-87-9) Hexane, 2,4-dimethyl (589-43-5) 3-Hexanone (589-38-8) Heptane, 4-methyl (589-53-7) Acetoin (513-86-0) Propanoic acid (79-09-4) Toluene (108-88-3) Octane (111-65-9) Hexane, 2,3,5-trimethyl (1069-53-0) Heptane, 2,4-dimethyl (2213-23-2) Butanoic acid, ethyl ester (105-54-4) Hexanal (66-25-1) 2,4-Dimethyl-1-heptene (19549-87-2) Heptane, 2,3-dimethyl (3074-71-3) Octane, 4-methyl (2216-34-4) Benzene, 1-ethynyl-4-methyl (766-97-2) Heptane, 2,4,6-trimethyl (2613-61-8) Butanoic acid, 2-methyl-, ethyl ester (7452-79-1) Ethylbenzene (100-41-4) Nonane (111-84-2) p-Xylene (106-42-3) 3-Hexen-1-ol (928-96-1) Pyrazine, 2,6-dimethyl (108-50-9) Pyrazine, ethyl- (6924-68-1) Benzene, 1-methyl-4-(1-methylethenyl)- (1195-32-0) Benzonitrile. 4-methyl (104-85-8) Heptane, 3,3,5-trimethyl (7154-80-5) Nonanal (124-19-6) Hexanoic acid, ethyl ester (123-66-0) Benzaldehyde (100-52-7) 3-Hexen-1-ol, acetate, (E)- (3681-82-1) Pyrazine, 2-ethyl-6-methyl- (13925-03-6) Acetic acid, hexyl ester (142-92-7) Pyrazine, trimethyl (14667-55-1) Octanal (124-13-0) D-Limonene (5989-27-5) p-Cymene (99-87-6) Decane, 3,7-dimethyl (17312548) Decane, 3,6-dimethyl (17312-53-7) Acetylpyrazine (22047-25-2) Ethanone, 1-(2-pyridinyl)- (1122-62-9) Phenol (108-95-2) 1,2-Cyclopentanedione, 3-methyl (765-70-8) Pyrazine, tetramethyl (1124-11-4) o-Isopropenyltoluene (7399-49-7) Butanoic acid, 3-methyl, 3-methylbutyl ester (106-27-4)) Nonanal (124-19-6) Heptadecane, 8-methyl (13287-23-5) Pyrazine, 2-methyl-3-(methylthio)- (2882-20-4) Decanal (112-31-2) Dodecane, 4-methyl (6117-97-1) Octane, 5-ethyl-2-methyl (62016186) Hexadecane, 2,6,10,14-tetramethyl (638-36-8)	HS-SPME-GC–MS	[56]

# Gas chromatography coupled with ion mobility spectrometry (GCxIMS) + solid-phase microextraction (SPME)–high-resolution direct analysis in real time AccuTOF™ mass spectrometry.

**Table 2:** Summary of key studies on the analysis of e-liquids and aerosols using liquid analysis.

Matrix Analyzed	Number of Compounds	Identified or Quantified Compounds (CAS)	Instrumental Technique	Reference
E-liquids	17	CBD (13956-29-1), Δ9-tetrahydrocannabinol (Δ9-THC) (1972-08-3), cannabinol (CBN) (521-35-7), cannabidiolic acid (CBDA) (1244-58-2), Δ9-tetrahydrocannabinolic acid A (Δ9-THCA) (23978-85-0), Δ8-tetrahydrocannabinol (Δ8-THC) (5957-75-5), cannabinol (CBN) (521-35-7), cannabigerol (CBG) (25654-31-3), cannabichromene (CBC) (20675-51-8), Cannabicyclol (CBL) (21366-63-2), cannabidivarin (CBDV) (24274-48-4), tetrahydrocannabivarin (THCV) (31262-37-0), cannabicitran (CBT) (31508-71-1), tetrahydrocannabivarinic acid (THCVA) (39986-26-0), cannabinolic acid (CBNA) (2808-39-1), cannabigerolic acid (CBGA) (25555-57-1), and cannabidivarinic acid (CBDVA) (31932-13-5)	LC-HRAM-MS and LC-UV	[98]
E-liquids and aerosols	8	N-nitrosonornicotine (NNN) (80508-23-2), N′-nitrosoanatabine (NAT) (887407-16-1), 4-(methylnitrosamino)-1-(3-pyridyl)-1-butanone (NNK) (64091-91-4), N-nitrosoanabasine (NAB) (37620-20-5), 4-(methylnitrosamino)-4-(3-pyridyl)-1-butanol (iso-NNAL) (59578-66-4), 4-(methylnitrosamino)-1-(3-pyridyl)-1-butanol (NNAL) (76014-81-8), 4-(methylnitrosamino)-4-3-pyridyl) butyric acid (iso-NNAC) (123743-84-0), and 4-(methylnitrosamino)-4-(3-pyridyl)-butanal (NNA) (64091-90-3)	UPLC-QTOF-HRMS	[99]
E-cigarette refill solutions	42	2-acetylpyrazine (22047-25-2), 2-acetylpyridine (1122-62-9), 2-acetylpyrrole (1072-83-9), 2-isopropyl-4-methylthiazole (15679-13-7), 2-methylpyrazine (109-08-0), 2,5-dimethylpyrazine (123-32-0), 2,6-dimethylpyridine (108-48-5), 2,3,5-trimethylpyrazine (14667-55-1), 2,3,5,6-tetramethylpyrazine (1124-11-4), 3-ethylpyridine (108-99-6), 3-methyl-3-phenylglycidate (77-83-8), 4-methyl acetophenone (122-00-9), 5-methylfurfural (620-02-0), carvone (6485-40-1), cocoa (8002-31-1), diethyl malonate (105-53-3), diethyl succinate (123-25-1), ethyl acetoacetate (141-97-9), ethyl cinnamate (103-36-6), ethyl lactate (687-47-8), ethyl phenylacetate (101-97-3), ethyl vanillin (121-32-4), ethyl-2-methylbutyrate (7452-79-1), ethyl 3-(methylthio)propionate (13327-56-5), ethyl maltol (4940-11-8), furaneol (3658-77-3), geraniol (106-24-1), ionone α (127-41-3), ionone β (14901-07-6), linalool (78-70-6), linalool oxide (60047-17-8), maltol (118-71-8), menthol (2216-51-5), menthone (10458-14-7), methyl cinnamate (103-26-4), methyl cyclopentenolone (765-70-8), methyl heptanone (110-93-0), methyl salicylate (119-36-8), nerol (106-25-2), nicotine (65-31-6), pyridine (110-86-1), vanillin (121-33-5), β-damascone (35044-68-9), γ-valeroactone (108-29-2), and γ-hexalactone (695-06-7)	HPLC-ESI–MS/MS	[100]
E-liquid samples	4	Tetrahydrocannabinol (THC) (1972-08-3), Mephedrone (1189805-46-6), Cumy-PeGaClone (Synthetic Cannabinoid) (2160555-55-3), and Methamphetamine (537-46-2)	DART-Q-TOF-MS/MS	[101]
E-cigarette fluids	4	Δ8-THC (5957-75-5), Δ9-THC (1972-08-3), CBD (13956-29-1), and CBN (521-35-7)	LC-MS/MS	[102]
E-liquid samples	21	Δ8-THC (5957-75-5), Δ9-THC (1972-08-3), Δ10-THC (95543-62-7), Δ6a,10a-THC (95720-02-8), CBDA (1244-58-2), THCA-A (23978-85-0), HHC (36403-90-4), Δ9-THCB (60008-00-6), Δ9-THCP (6465-30-1), Δ8-THCP (5957-75-5), Δ9-THCH (81586-39-2), cannabicitran (CBT) (31508-71-1), CBG (25654-31-3), CBN (521-35-7), CBD (13956-29-1), CBC (20675-51-8), CBDV (24274-48-4), acetate ester of Δ9-THC (885123-57-9), acetate ester of CBD (23050-54-6), acetate ester of CBN (51895-51-3), and acetate ester of HHC (not reported).	LC-MS/MS	[103]

**Table 3:** Analytical techniques and matrices for PTM determination in vaping products.

Matrix Analyzed	Analytes (Range)	Instrumental Technique	Reference
3 brands of e-liquids (residual)	Cd (0.035 µg/g) Co (0.51–0.55 µg/g) Cr (3.22–5.33 µg/g) Cu (3.50–194.6 µg/g) Ni (10.90–12.42 µg/g) Pb (0.15–0.61 µg/g)	ICP OES	[143]
E-liquid refills within e-cigarettes	Cd (<LQ) Cr (0.033–0.396 µg/g) Cu (9.18–903 µg/g) Ni (0.040–4.04 µg/g) Pb (<LQ) Sn (0.119–1.35 µg/g) Zn (3.00–454 µg/g)	ICP-MS	[144]
E-liquids and aerosols from pod-based devices from three manufacturers	Cd (0.100–0.108 µg/g) Cr (0.025–1.64 µg/g) Cu (2.00–927 µg/g) Ni (0.025–61.3 µg/g) Pb (0.066–2.56 µg/g) Sn (0.099–58.2 µg/g) Zn (1.00–14.9 µg/g)	ICP-MS	[145]
Aerosols emitted from 12 brands of e-liquids	Cd (<LOD) Cr (0.231–1.85 ng/10 puffs) Cu (2.53–488 ng/10 puffs) Ni (0.472–9.63 ng/10 puffs) Pb (0.128–11.4 ng/10 puffs) Sn (0.341–1.71 ng/10 puffs) Zn (27.9–339 ng/10 puffs)	ICP-MS	[146]
22 e-liquid refills and their constituents	Cd (0.001–0.141 µg/g) Cr (0.003–0.036 µg/g) Cu (0.004–0.055 µg/g) Ni (0.002–0.092 µg/g) Pb (0.001–0.011 µg/g) As (ND)	TXRF	[147]
Aerosols emitted from 8 brands of e-liquids (chemical speciation of arsenic)	iAS^III^ (1.06–4.66 µg/kg) iAS^V^ (0.71–8.30 µg/kg) MMA (0.05–2.28 µg/kg)	HPLC-ICP-MS	[148]
Aerosols emitted from 50 brands of e-liquids	Cd (0.01–1.60 ng/m^3^) Cr (0.41–126.17 ng/m^3^) Ni (0.03–4.49 ng/m^3^) Pb (0.06–7.88 ng/m^3^)	ICP-MS	[149]
Aerosols emitted from 4 synthetic e-liquids	Cu (0.0001–8.3 mg/L) Fe (0.07–55.0 mg/L) Ni (0.08–24.0 mg/L) Pb (0.04–5.8 mg/L) Zn (0.02–22.0 mg/L)	TXRF	[150]
Aerosols emitted from 16 brands of e-liquids and 4 e-cigarettes	Al (5.39–34.7 µg/kg) As (0.11–7.76 µg/kg) Cd (0.06–1.98 µg/kg) Cr (0.06–39.4 µg/kg) Cu (6.3–1936 µg/kg) Fe (4.44–200 µg/kg) Mn (0.42–96.1 µg/kg) Ni (8.00–2491 µg/kg) Pb (6.50–1079 µg/kg) Sb (0.06–13.2 µg/kg) Sn (0.51–322 µg/kg) U (0.04–0.06 µg/kg) W (0.05–0.15 µg/kg) Zn (387–6952 µg/kg)	ICP-MS	[151]
Aerosols emitted from 16 brands of e-liquids and 4 e-cigarettes	Al (6.02–16.7 µg/kg) As (0.09–2.07 µg/kg) Cd (0.04–0.16 µg/kg) Cr (0.05–7.99 µg/kg) Cu (15.4–615 µg/kg) Fe (3.46–194 µg/kg) Mn (0.77–51.4 µg/kg) Ni (0.73–1181 µg/kg) Pb (2.38–377 µg/kg) Sb (0.33–4.29 µg/kg) Sn (0.37–208 µg/kg) U (0.04–0.06 µg/kg) W (0.04–0.25 µg/kg) Zn (704–3420 µg/kg)	ICP-MS	[152]

**Table 4:** Estimated daily exposure to PTMs from vaping compared to regulatory limits.

Metal	ICH (µg/day)	ATSDR (µg/m^3^)	NIOSH (mg/m^3^)	NIOSH Daily Value (mg)
Cd	3	0.03	0.005	0.03
Cr	3	NI	0.5	3.3
Cu	30	NI	1.0	6.7
Ni	6	0.09	0.015	0.1
Pb	5	NI	0.03	0.2
Zn	NI	NI	5.0	33.3

NI: Not informed.

## Data Availability

The data presented in this study are available on request from the corresponding author.

## Abbreviations

The following abbreviations are used in this manuscript:

PAHs	Polycyclic aromatic hydrocarbons
TSNAs	Tobacco-specific nitrosamines
THC	Tetrahydrocannabinol
CAGR	Compound annual growth rate
GC-MS	Gas chromatography–mass spectrometry
HPLC	High-performance liquid chromatography
ICP	Inductively coupled plasma
EU	European Union
TPD	Tobacco Products Directive
MHRA	Medicines and Healthcare Products Regulatory Agency
GABA	Gamma-AminoButyric Acid
TRPV1	Transient Receptor Potential Vanilloid 1
TRPA1	Transient Receptor Potential Ankyrin 1
GRAS	Generally Recognized as Safe
EVALI	e-cigarette or vaping product use-associated lung injury
DCP	Disease Control and Prevention
CBD	Cannabidiol
BAL	Bronchoalveolar lavage
PG	Propylene glycol
GLY	Glycerin
DnS	Dilute-and-shoot
SPE	Solid-phase extraction
LLE	Liquid–liquid extraction
SPME	Solid-phase microextraction
GCxIMS	Chromatography coupled with ion mobility spectrometry
LC–MS/MS	Liquid chromatography coupled with tandem mass spectrometry
LC-HRAM-MS	Liquid chromatography coupled with high-resolution accurate mass spectrometry
LC-UV	Liquid chromatography coupled with ultraviolet detection
UPLC-QTOF-HRMS	Quadrupole time-of-flight high-resolution mass spectrometry
EPA	Environmental Protection Agency
GC-FID	Gas chromatography with flame ionization detection
GC-TCD	Gas chromatography with thermal conductivity detection
ICP-MS	Inductively coupled plasma mass spectrometry
TXRF	Total reflection X-ray fluorescence
ICP-OES	Inductively coupled plasma optical emission spectrometry
RM	Reference material
LOD	Limit of detection
SEM	Scanning electron microscopy
WHO	World Health Organization
ICH	International Council for Harmonization of Technical Requirements for Pharmaceuticals for Human Use
ATSDR	Agency for Toxic Substances Disease Registry
NIOSH	National Institute for Occupational Safety and Health
LIBS	Laser-Induced Breakdown Spectroscopy
EIS	Electrochemical Impedance Spectroscopy
DPASV	Differential Pulse Anodic Stripping Voltammetry
SWASV	Square Wave Anodic Stripping Voltammetry
CV	Cyclic Voltammetry
2D-IR	Two-Dimensional Infrared Spectroscopy
